# Not all light transmission aggregation assays are created equal: qualitative differences between light transmission and 96-well plate aggregometry

**DOI:** 10.1080/09537104.2018.1466388

**Published:** 2018-05-01

**Authors:** Melissa V. Chan, Philip D. Leadbeater, Steve P. Watson, Timothy D. Warner

**Affiliations:** 1 The Blizard Institute, Barts & The London School of Medicine and Dentistry, Queen Mary University of London, London, UK; 2 Intensive Care Unit, Western General Hospital, Edinburgh, UK; 3 Institute of Cardiovascular Sciences, College of Medical and Dental Sciences, University of Birmingham, Birmingham, UK

**Keywords:** 96-well, light transmission, platelet reactivity

## Abstract

In this short article, submitted as part of the review on platelet function testing, we illustrate the quantitative and qualitative differences between classical light transmission aggregometry (LTA) and 96-well plate aggregometry.

We show that responses to platelet agonists and antagonists differ depending upon the method of aggregation testing. For example, in 96-well aggregometry, responses to collagen are strongly inhibited by P2Y_12_ receptor antagonists while in LTA they are much less affected. Furthermore, we explore the importance of differences in the mechanical environment upon platelet aggregation.

We emphasize that LTA and 96-well aggregometry are not interchangeable assays. These two assays are best used as complementary tests to explore platelet function in depth.

## Introduction

First reported over 50 years ago (1), light transmission aggregometry (LTA) is still considered as the gold standard way to measure platelet reactivity. It relies upon the simple principle that as a suspension of platelets aggregates, the transmission of light through the suspension increases allowing one to follow platelet aggregation in real time. In addition, it has the advantage (which was not realized when first described) of being able to record the preceding change in shape of platelets from a discoid to a spherical structure (seen as an increase in optical density of the suspension).

To perform this assay in standard light transmission aggregometers, 200–500 µl suspensions of platelets in plasma (platelet-rich plasma, PRP) or in buffer (washed platelets) are placed in warmed cuvettes containing a magnetic stirrer bar and typically stirred at 1200rpm (in some aggregometers the rate can be varied) before addition of test compounds. The aggregation of platelets is then followed by measurement of the change in transmission of light shone across the cuvette. This traditional LTA has many advantages: it is well established, shows the kinetics of platelet responses (including shape change and reversible aggregation), is relatively inexpensive, and results can be compared to 50 years of existing data. However, LTA is quite laborious to run, is not a point-of-care test, is considered by some but not all researchers to be variable and poorly reproducible, and is therefore operator-dependent (with notable variability between different test centers) and there are significant discrepancies in data analysis and interpretation (2). Furthermore, the degree of light transmission not only reflects aggregation but is influenced by platelet granule secretion, and there are differences between the optical density of micro- and macro-aggregates.

In recent years, 96-well plate readers have become standard laboratory equipment and they measure absorbance that can be used to follow changes in the transmission of light through suspensions of platelets, making them attractive alternatives to traditional aggregometers. They have the significant advantage that they can measure up to 96 “channels” in parallel with small sample volumes (as little as 40 μl), making them much more efficient than traditional LTA machines, which generally have between 2 and 8 channels, and require greater sample volumes. To further simplify the assay, agonists can be added to the plate in advance of the platelet suspension, meaning that a multichannel pipette can be used to add platelets into the 96 wells at the same time. However, 96-well plate readers do not mix the platelets in the same way as traditional aggregometers and the readers cannot follow changes in absorbance concurrently with mixing. Therefore, unlike traditional LTA, 96-well plate aggregometry cannot be used as a kinetic assay. Furthermore, as the physical forces acting on platelets are key determinants of their responses to agonists, the data derived in the two assays are not interchangeable. We provide example data here to highlight the differences and discuss the best use of the two assays.

## Methods

Blood was collected into 3.2% sodium citrate (1:9) from healthy volunteers who had abstained from antiplatelet drugs for 2 weeks. The study was approved by the NHS St. Thomas’ Hospital Research Ethics Committee (07/Q702/24). PRP was obtained by centrifugation of whole blood (175*xg*, 15 min, 25°C) and platelet-poor plasma (PPP) was made by further centrifugation of PRP (12000*xg*, 2 min, 25°C). PRP was incubated with vehicle, cyclooxygenase (COX) inhibitor aspirin (ASA, 30 μM; Sigma-Aldrich, Poole, UK), or P2Y_12_ receptor antagonist AR-C66096 (1 μM; Tocris, Abingdon, UK) for 30 min at 37°C.

Aggregometry was assessed by LTA in a PAP-8E aggregometer (Bio/Data Corporation, Horsham, PA, USA) and in a 96-well full-area plate shaken in a thermoshaker (BioShake IQ, QUANTIFOIL Instruments, Jena, Germany). In LTA, 25 μl agonist was added to 225 μl PRP, while in 96-well aggregometry, 90 μl PRP was added to 10 μl agonist. In both cases, PRP was mixed at 1200rpm for 5 min at 37°C. Responses were generated to arachidonic acid (AA, 0.03–1 mM; Sigma-Aldrich, Poole, UK), adenosine diphosphate (ADP, 0.005–40 μM; Labmedics, Salford, UK), collagen (0.01–40 μg/ml, Takeda, Linz, Austria), and U46619 (0.005–40 μM; Cayman Chemical Company, Ann Arbor, USA). Light absorbance was converted to transmission to allow for direct comparison of data.

To explore the effects of different mixing regimes in other LTA experiments, PRP was stirred at 100, 300, and 1200rpm and stimulated with ADP (3 μM), collagen (3 μg/ml), or U46619 (3 μM), respectively.

## Results

### LTA and 96-well plate aggregometry

**Figure 1.**
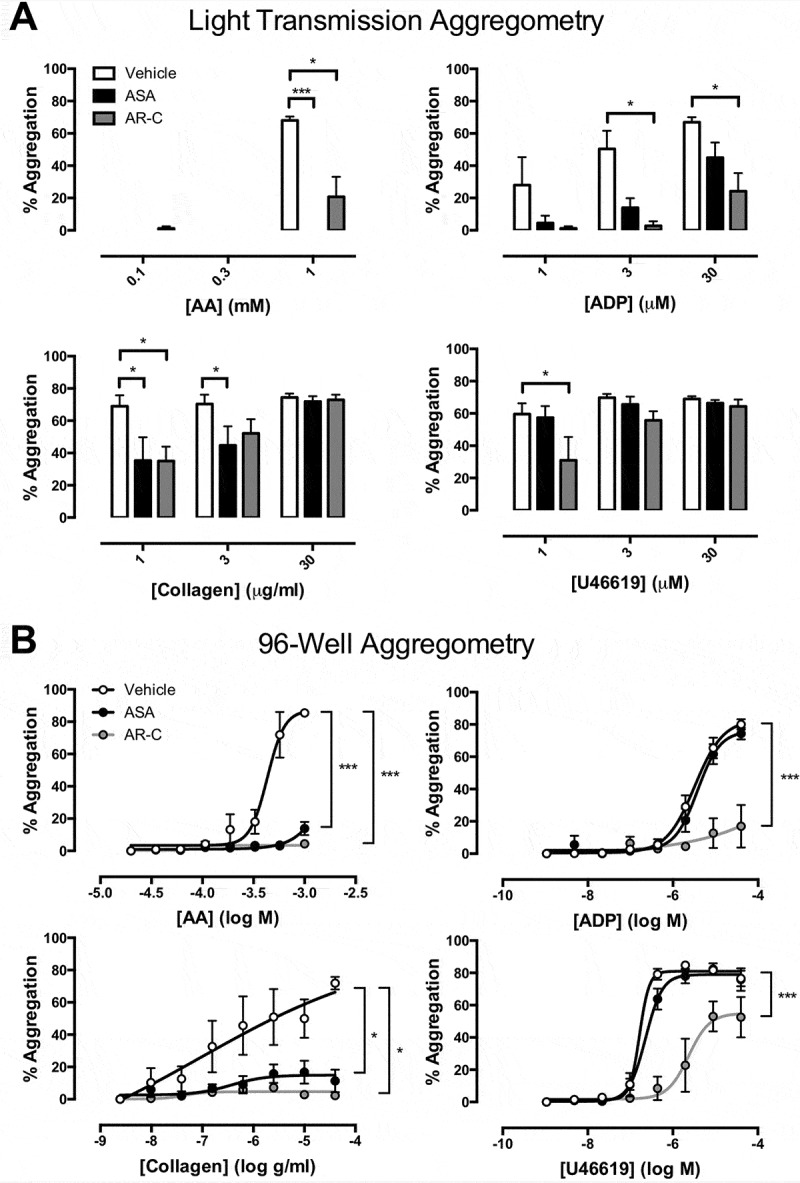
Final aggregation as measured by (A) light transmission aggregometry and (B) 96-well aggregometry in the presence of vehicle (white), aspirin (ASA, 30 μM; black), and AR-C66096 (AR-C, 1 μM; grey) in response to arachidonic acid (AA; 0.03–1 mM), ADP (0.005–40 μM, collagen (0.01–40 μg/ml), and U46619 (0.005–40 μM). Data were analyzed by one-way or two-way ANOVA and assessed as significant to *p < 0.05. *N* = 5 for each.

In 96-well aggregometry, aspirin (*p* < 0.001) and AR-C66096 (*p* < 0.001) strongly inhibited the responses to all concentrations of collagen (0.01–40 μg/ml). Responses were less affected in LTA; for example, aggregations in response to 30 μg/ml collagen were 74 ± 6% in the presence of vehicle, 72 ± 3% in the presence of aspirin, and 73 ± 3% in the presence of AR-C66096 (Figure 1A&B).

Aspirin had no effect against aggregations induced by ADP in 96-well aggregometry but in LTA, it abolished aggregations to low concentrations of ADP. For example, the aggregation to 1 μM ADP was reduced from 28 ± 17% to 5 ± 5% by aspirin and the aggregation to 3 μM ADP from 50 ± 11% to 14 ± 6% (*p* < 0.05, Figure 1A).

Aggregation to AA was blunted by AR-C66096 in LTA while it was abolished in 96-well aggregometry. Aggregations induced by U46619 were unaffected by aspirin in either LTA or 96-well aggregometry, but were substantially reduced by AR-C66096 (Figure 1B).

### Effect of stirring speed on aggregations in LTA

**Figure 2.**
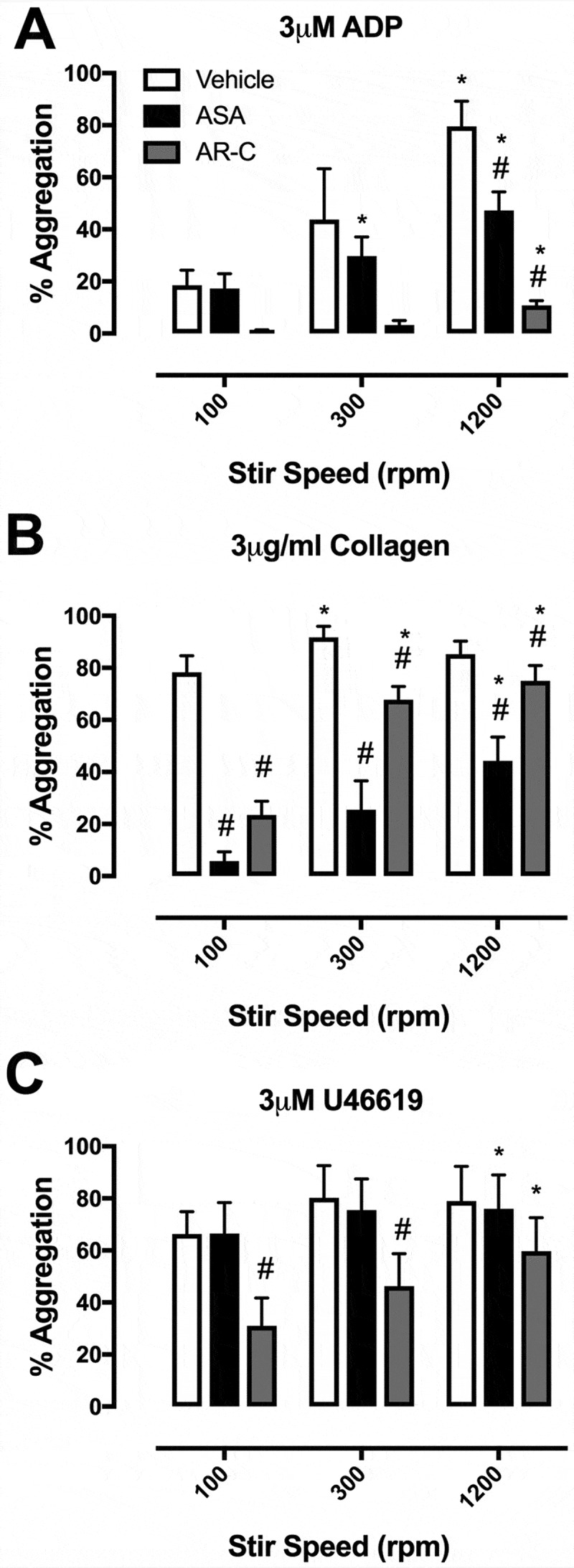
Final aggregation in light transmission aggregometry with stir speeds of 100, 300, and 1200 rpm in the presence of vehicle (white), aspirin (ASA, 30 μM; black), and AR-C66096 (AR-C, 1 μM; grey) in response to (A) ADP (3 μM), (B) collagen (3 μg/ml), and (C) U46619 (3 μM). Data were analyzed by one-way ANOVA and assessed as significant to *p* < 0.05; * vs 100 rpm and $ vs vehicle. *N* = 5 for each.

In LTA, 3 μM ADP caused 19 ± 6% aggregation in platelets stirred at 100rpm, 44 ± 20% in platelets stirred at 300rpm, and 80 ± 10% in platelets stirred at 1200rpm. Aggregations to 3 μg/ml collagen and 3μM U46619 were similarly increased with increased stirring speed (Figure 2). At 100 rpm, aggregation to collagen was strongly inhibited by aspirin and AR-C66096 but less so at higher stir speeds (Figure 2).

## Discussion

Here using identical preparations of platelets, platelet agonists, and inhibitors, we demonstrate notable differences in responses obtained in LTA versus 96-well plate aggregometry. As all other parameters were identical these differences can only be explained by differences in the mechanical/physical environments of the two assays (3). LTA is fairly standardized with respect to the mixing of platelets with a stirrer bar turning at 1000–1200rpm. However, kinetic 96-well plate readers which repeatedly mix and read samples at fixed intervals (often 10–15 s) each have their own mixing patterns, generally planar (4), orbital, or high-speed shaking (5); so data from different plate readers cannot be directly compared. For this reason, we have chosen a stand-alone 96-well plate mixer that produces individual vortices in each of the 96 wells, somewhat akin to the mixing seen with a stirrer bar in a standard aggregometer. We have coupled this with half-area 96-well plates pre-coated with a standardized panel of agonists where the operator needs only to pipette PRP and PPP into the appropriate wells. Aggregation can then be calculated easily from a set formula (6,7). Furthermore, the large number of replicates and broad range of agonist concentrations allow 96-well plate aggregometry to be used to construct concentration-response curves, which permits deeper analyses of platelet function, useful to the characterization of the effects of antiplatelet drugs or genetic variations. While this is a standardized assay, we have previously demonstrated how mixing the 96-well plates within a kinetic plate reader produces different results than mixing the same plates on the external vortex mixer (7), another illustration of the strong influences of the mechanical environment upon platelet aggregation.

One of the notable differences we found between LTA and 96-well aggregometry was that the effects of aspirin and P2Y_12_ receptor blockade were much more marked in 96-well aggregometry than in LTA. In particular, AR-C66096 inhibited aggregations to collagen at all concentrations tested in 96-well aggregometry, but only at the lowest collagen concentration in LTA. Similarly, it has been previously shown that clopidogrel has greater effects upon collagen-induced aggregation measured in 96-well aggregometry than in LTA (8,9). We can speculate that differences in the volume of PRP and the presence or not of a stir bar play a critical role in determining fluid dynamics within the sample and the formation of aggregates dependent upon secondary mediators. In further experiments, we found that simply reducing the speed of LTA from the usual 1200 rpm to 300 rpm or 100 rpm produces marked changes in the apparent potency of platelet agonists and the efficacies of antiplatelet drugs. At higher stir speeds, the chance of platelet-platelet interactions is increased but, conversely, the greater the stirring speed, the higher the forces will be to break up any weaker platelet aggregates. Indeed, since we see different effects of stirring speed upon the efficacies of aspirin and AR-C66096, it may well be that the secondary mediators, thromboxane A_2_ and ADP, play distinctive roles in the presence of different forces. As different 96-well kinetic plate readers have different mixing forces, it can be readily appreciated that the 96-well plate aggregometry can be less standardized than traditional LTA, which itself is well-known to vary between laboratories and individual operators.

In summary, 96-well plate aggregometry is best used with standardized mixing external to the 96-well plate reader and the construction of multiple concentration-response curves to a range of agonists to provide broad phenotyping of platelet responses; LTA is best used to deeply phenotype the kinetics of the platelet responses to individual concentrations of agonists. Data from the two assays are not interchangeable but are complementary.

## References

[1] Born GVR. Aggregation of blood platelets by adenosine diphosphate and its reversal. Nature. 1962;194:927–929. doi:10.1038/194927b0. 13871375

[2] Cattaneo M, Cerletti C, Harrison P, Hayward CPM, Kenny D, Nugent D, et al Recommendations for the standardization of light transmission aggregometry: a consensus of the working party from the platelet physiology subcommittee of SSC/ISTH. J Thromb Haemost. 2013;11:1183–1189. doi:10.1111/jth.12231. 23574625

[3] Lordkipanidzé M, Lowe GC, Kirkby NS, Chan MV, Lundberg MH, Morgan NV, et al Characterization of multiple platelet activation pathways in patients with bleeding as a high-throughput screening option: use of 96-well Optimul assay. Blood. 2014;123:e11–e22. doi:10.1182/blood-2013-08-520387. 24408324 PMC3931193

[4] Fratantoni JC, Poindexter BJ Measuring platelet aggregation with microplate reader. A new technical approach to platelet aggregation studies. Am J Clin Pathol. 1990;94:613–617. doi:10.1093/ajcp/94.5.613. 2239825

[5] Peace AJ, Tedesco AF, Foley DP, Dicker P, Berndt MC, Kenny D Dual antiplatelet therapy unmasks distinct platelet reactivity in patients with coronary artery disease. J Thromb Haemost. 2008;6:2027–2034. doi:10.1111/j.1538-7836.2008.03157.x. 18823340

[6] Chan MV, Armstrong PCJ, Papalia F, Kirkby NS, Warner TD Optical multichannel (optimul) platelet aggregometry in 96-well plates as an additional method of platelet reactivity testing. Platelets. 2011;22:485–494. doi:10.3109/09537104.2011.592958. 21806492

[7] Chan MV, Warner TD Standardised optical multichannel (optimul) platelet aggregometry using high-speed shaking and fixed time point readings. Platelets. 2012;23:404–408. doi:10.3109/09537104.2011.603066. 21806495

[8] Dyszkiewicz-Korpanty A, Olteanu H, Frenkel EP, Sarode R Clopidogrel anti-platelet effect: an evaluation by optical aggregometry, impedance aggregometry, and the Platelet Function Analyzer (PFA-100^TM^). Platelets. 2007;18:491–496. doi:10.1080/09537100701280654. 17852774

[9] Armstrong PC, Dhanji AR, Truss NJ, Zain ZN, Tucker AT, Mitchell JA, et al Utility of 96-well plate aggregometry and measurement of thrombi adhesion to determine aspirin and clopidogrel effectiveness. Thromb Haemost. 2009;102:772–778. 19806265 10.1160/TH09-04-0215

